# Supplementary material for: Application of C5.0 Algorithm for the Assessment of Perceived Stress in Healthcare Professionals Attending COVID-19
Source: Brain Sci. 2023 Mar 20;13(3):513. doi: 10.3390/brainsci13030513 (PMC10046351; doi:10.3390/brainsci13030513)
Supplement: Supplementary file 1 [file brainsci-13-00513-s001.zip › brainsci-2263081-supplementary.pdf]

Figure S1 shows the box plot built upon the five areas of the ACSS, derived from the calculation of the central tendency metrics for each of these components based on the cumulative result of each participant.

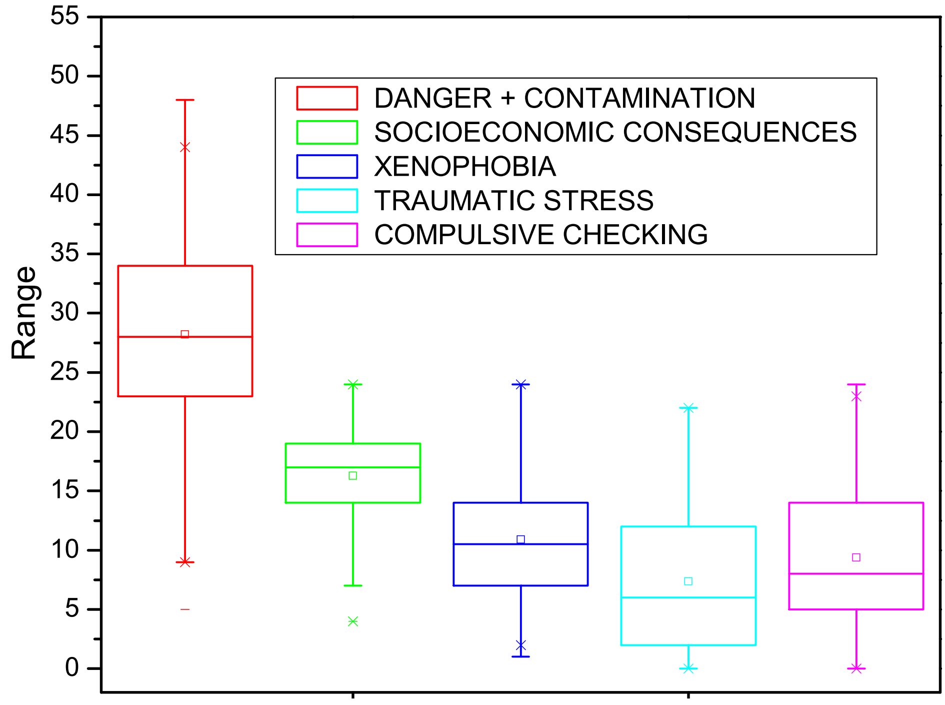

Figure S1. Central tendency metrics for each of the ACSS components.

We implemented an evaluation of performance of a subset of supervised learning algorithms as shown in Table S1. When evaluating the results *k*NN showed to have the best results for this dataset, nevertheless, the need for these results to be significant in the clinical practice requires to have an explicable result in such a way that the clinician is provided with a set of rules or steps to facilitate decision making and, thus, incorporate this approach as a computer assisted diagnosis. For the previous reasons, a decision tree was selected as the ideal algorithm for this situation, even though it is relatively simple and did not show the best performance results from a machine learning perspective. Still, when paired with the needs in the clinical practice a Decision Tree became the viable option to approach this challenge.

Table S1 Performance Evaluation of Algorithms Explored

Algorithm	AUC	CA	F1	Precision	Recall
kNN	0.956	0.892	0.890	0.895	0.892
SVM	0.909	0.735	0.689	0.684	0.735
SGD	0.688	0.696	0.654	0.621	0.696
Random Forest	0.918	0.814	0.806	0.819	0.814
Multilayer Perceptron	0.863	0.765	0.751	0.754	0.765
Naïve Bayes	0.932	0.578	0.627	0.841	0.578
Logistic Regression	0.810	0.686	0.670	0.671	0.686
Classification Tree	0.716	0.676	0.661	0.647	0.676
AdaBoost	0.842	0.804	0.807	0.821	0.804

kNN = *k*-nearest neighbors
SVM= support vector machine
SGD= Stochastic gradient descent
